# New Insights into Adiponectin and Leptin Roles in Chronic Kidney Disease

**DOI:** 10.3390/biomedicines10102642

**Published:** 2022-10-20

**Authors:** Susana Coimbra, Susana Rocha, Maria João Valente, Cristina Catarino, Elsa Bronze-da-Rocha, Luís Belo, Alice Santos-Silva

**Affiliations:** 1 TOXRUN—Toxicology Research Unit, University Institute of Health Sciences, Cooperativa de Ensino Superior Politécnico e Universitário (CESPU), CRL, 4585-116 Gandra, Portugal; 2 Associate Laboratory i4HB-Institute for Health and Bioeconomy, Faculdade de Farmácia da Universidade do Porto, 4050-313 Porto, Portugal; 3 UCIBIO—Applied Molecular Biosciences Unit, Department of Biological Sciences, Faculdade de Farmácia da Universidade do Porto, 4050-313 Porto, Portugal; 4 National Food Institute, Technical University of Denmark, 2800 Kongens Lyngby, Denmark

**Keywords:** adiponectin, leptin, chronic kidney disease, cardiovascular disease, inflammation, comorbidities

## Abstract

Chronic kidney disease (CKD) is commonly associated with a high burden of comorbidities and poor clinical outcomes. Malnutrition–inflammation–atherosclerosis syndrome is common in the more severe stages of CKD, suggesting a close interplay for these three comorbid conditions. Both malnutrition and obesity are associated with a disturbed adipokine profile and inflammation, contributing to a higher risk of cardiovascular disease (CVD) events. Adiponectin and leptin have important roles in carbohydrate and lipid metabolism, and in the inflammatory process. The effects of adiponectin and leptin alterations in CKD, which are usually increased, and their association with the different comorbidities found in CKD, will be focused on to understand their crosstalk with the risk of CVD events. Nonetheless, although adiponectin and leptin contribute to a higher risk of CVD events, further studies are warranted to fully clarify their roles, especially when different comorbidities exist.

## 1. Introduction

Chronic kidney disease (CKD) is a long-term condition characterized by a gradual loss of kidney function. End-stage renal disease (ESRD), the worst stage of CKD, is a major public health problem, given the increasing prevalence worldwide and its socioeconomic consequences. Moreover, worsening of CKD is associated with a high burden of comorbidities.

Different structural and functional changes occur within the kidney during the onset of the disease, leading to glomerular, tubular and vascular injuries [1]. The progression is accompanied by renal inflammation, hypoxia and oxidative stress, which leads to progressive renal fibrosis, and to other functional adaptations to renal damage, triggering different pathophysiological processes within the kidneys and in other organs and systems [2]. The low-grade inflammation in the early stages of CKD increases with the worsening of the disease, and thereby contributes towards magnifying other features of the uremic phenotype, such as atherosclerosis, depression, protein–energy wasting, vascular calcification and heart-related complications. Inflammation can be a trigger or a consequence of CKD; it may result from the primary cause of CKD, such as diabetes, hypertension and obesity (leading to renal damage), or can be triggered by associated renal dysfunction changes (e.g., uremia, oxidative stress, metabolic acidosis) [3,4]. In fact, diabetes mellitus and hypertension are the most common causes of CKD, and are recurrent comorbidities in this condition. Recent studies on the pathophysiology of obesity-related CKD indicate that the low-grade inflammation and the abnormal lipid metabolism in obese individuals contribute to kidney cell injury, increasing the risk of developing CKD [5]. Moreover, a higher prevalence of protein–energy malnutrition, atherosclerosis and cardiovascular disease (CVD) events have been associated with worsening of CKD, suggesting a close interplay of these three comorbid conditions with CKD severity. Thus, paradoxically, both malnutrition and obesity, associated with inflammation, seem to lead to a higher risk of CVD events and suggest a particular role for the adipose tissue, namely, the altered production of adipokines. Indeed, mortality and morbidity risk in CKD patients, especially in ESRD patients, is high, and CVD events are the most common causes of death [6].

Adiponectin and leptin are two adipokines that present important roles in carbohydrate and lipid metabolism, as well as in the inflammatory process (Table 1). Adiponectin shows cardioprotective and anti-inflammatory effects, while leptin is associated with several obesity-related CVDs and with inflammatory activity. Again, paradoxically, in CKD patients, adipokines, adiponectin and leptin, and the ratio leptin/adiponectin, are increased, independently of traditional renal disease risk factors [7]. Although known as important mediators of cardiometabolic risk in obesity, the role of these adipokines in CKD, namely in ESRD patients, is poorly clarified.

Dyslipidemia is known as one of the major traditional risk factors for CVD in the general population, and the same occurs in CKD patients. Comorbidities, such as vascular calcification, left ventricular hypertrophy (LVH), anemia, mineral and bone disorder, are also risk factors for an adverse outcome in CKD patients, especially in ESRD patients. Interestingly, it has been reported that ESRD patients have protective normal or lower levels of total cholesterol and low-density lipoprotein cholesterol (LDLc), along with non-protective lower high-density lipoprotein cholesterol (HDLc) and higher triglyceride values. The prevalence and severity of CVD events are high, as compared to the general population, and difficult to explain, when considering the traditional CVD risk factors [8].

We believe that in ESRD patients, other non-conventional factors, including inflammatory biomarkers, dyslipidemia and adipocyte-related cytokines, might contribute to the inappropriately high rate of CVD events. It is known that the adipose tissue is a complex organ with other functions beyond the storage of energy, for example, the secretion of adipokines and the production of inflammatory cytokines, such as tumor necrosis factor (TNF) and interleukin (IL)-6. These molecules with multiple effects are able to modulate different signaling pathways, such as insulin signaling, endothelial health and other CVD risk factors. The uremic milieu may, in fact, alter the normal adipocyte metabolism, leading to pathogenic effects. The changes and relationships of adipokines with the different players in CKD will be focused on in an attempt to understand their crosstalk with CVD risk factors.

## 2. Adiponectin

In CKD patients, adiponectin production is increased in the visceral and subcutaneous adipose tissues, and its blood levels are high and particularly enhanced in ESRD patients. Nevertheless, circulating adiponectin levels are inversely related to body mass index (BMI) values in ESRD patients [9]. Despite the anti-inflammatory, antidiabetic and antiatherogenic properties of adiponectin, the development of insulin resistance, systemic inflammation and CVD are common in these patients. It is still unclear whether its effects on metabolism are disturbed in CKD patients, considering their higher levels of adiponectin.

Adiponectin, a 30 kDa protein hormone mainly produced by adipocytes, comprising 244 amino acids, is composed of a N-terminal collagen-like sequence (collagen domain) and a C-terminal globular region (globular domain). Three different isoforms have been identified: trimer (low-molecular weight), hexamer (middle-molecular weight) and multimer (high-molecular weight, HMW) adiponectin. In blood, adiponectin circulates in the globular form as trimer, hexamer and multimer isoforms. The latter, i.e., the HMW adiponectin, appears the most biologically active [10]. Adiponectin isoform levels can be determined by several methods, based on velocity gradient centrifugation, polyacrylamide gel electrophoresis, gel filtration chromatography and enzyme-linked immunosorbent assays [11].

Adiponectin mediates its functions through three receptors: adiponectin receptor (AdipoR)1 (predominant in skeletal muscles), AdipoR2 (predominant in the liver) and T-cadherin [10]. Globular and trimeric adiponectin interact predominantly with AdipoR1 and AdipoR2, whereas hexamers and multimers interact mainly with T-cadherin receptors.

Adiponectin has several metabolic functions, including suppressing hepatic glucose production and increasing cellular glucose uptake and insulin sensitivity. It is also involved in lipid metabolism, enhancing skeletal muscle fatty oxidation and suppressing lipid accumulation in the liver [12]. The interaction of adiponectin with AdipoR1 regulates glucose metabolism, insulin sensitivity and fatty acid oxidation through the activation of the 5′-AMP-activated protein kinase (AMPK) pathway [13]; the interaction with AdipoR2 induces the activation of peroxisome proliferator-activated receptor (PPAR)-α, inducing fatty acid catabolism [14].

### 2.1. Inflammation and Adiponectin

The anti-inflammatory effects of adiponectin include the suppression of M2 to M1 macrophage differentiation and the inhibition of the production of several proinflammatory cytokines, such as TNF-α and IL-6. It is also able to reduce leptin production [15] and to protect cells from apoptosis [16].

Decreased circulating levels of adiponectin have been reported under inflammatory conditions typical of obesity, type 2 diabetes, metabolic syndrome and CVD [17]. An association between low levels of this adipokine and increased risk of atherosclerosis, the development of diabetes mellitus and CVD have been widely reported [17].

**Figure 1:**
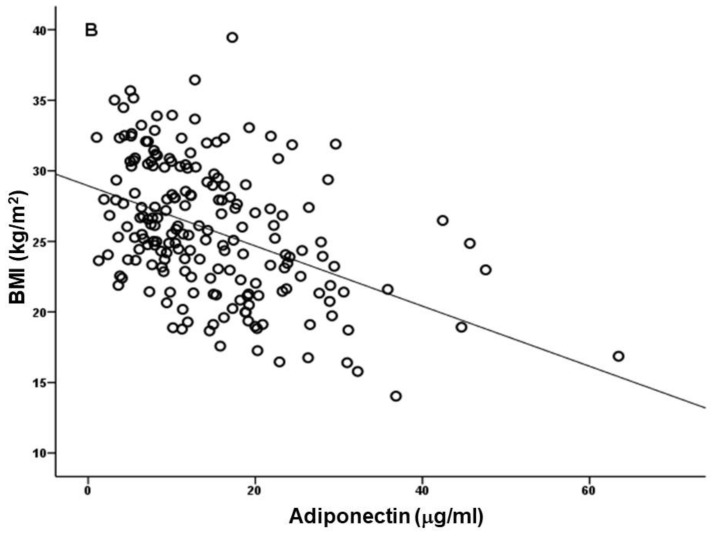
Association of circulating levels of adiponectin with body mass index (BMI) values in end-stage renal disease patients under hemodialysis (*r* = −0.431, *p* < 0.001). Adapted from Coimbra et al. 2019 [9].

Considering that inflammation is common in CKD, and increases with renal disease progression [18], being especially high in ESRD patients [9], one would expect to find lower adiponectin levels, as occurs in other inflammatory conditions. In spite of this, in CKD patients, adiponectin correlates inversely with body mass index (BMI; Figure 1); however, it shows a negative correlation with glomerular filtration rate (GFR) and a positive correlation with proteinuria, that is, a relationship with renal dysfunction [19]. High serum adiponectin has been proposed by some authors as a predictor of progression to ESRD in CKD patients; however, some data suggest that adiponectin may be a biomarker of renal dysfunction, instead of a risk factor of CKD progression [20].

The decreased renal excretion in CKD favors the increase in adiponectin circulating values [21]; however, the impaired renal clearance of adiponectin does not completely explain its rise. Several hypotheses have been raised to further explain the high adiponectin concentrations found in CKD.

One of these hypotheses is the development of adiponectin resistance, particularly in dialysis patients, as a consequence of the uremic milieu. Indeed, it was reported that in human tissues, AdipoR1 is upregulated by uremia, and that resistance occurs at the post-receptor level [22]. Another explanation is that adiponectin is highly secreted to counterbalance the effects of the high levels of inflammatory cytokines and/or vascular injuries; however, the proatherogenic uremic and inflammatory environments appear to overwhelm its effects [23]. It has also been hypothesized that adiponectin may present anti- or proinflammatory physiological roles. Conditions with moderate to severe inflammation, such as CKD, rheumatoid arthritis and inflammatory bowel disease, have been positively associated with high serum levels of adiponectin [15].

In addition to the uncertainty regarding the cause(s) of the higher circulating levels of adiponectin, its role in disease progression and CKD-related comorbidities is also a matter of debate, and the physiological roles of adiponectin isoforms need further studies.

### 2.2. CVD Risk and Adiponectin

In CKD, the relationship between the occurrence of CVD events and the high adiponectin levels is also still polemic.

#### 2.2.1. BMI

In a retrospective analysis, high circulating adiponectin levels were associated with increased mortality in CKD patients with low BMI, at stages 3 to 4 [24]. According to the authors, a 1 μg/mL increase in adiponectin was associated with a 3% increased risk of all-cause and 6% increased risk of CV mortality [24]. Rhee et al. reported that higher adiponectin was associated with a threefold higher death risk in dialysis patients, independently of body composition and lipid levels [25].

Indeed, it was reported that in CKD, a better physical health-related quality of life is associated with low circulating adiponectin levels [26]. The high adiponectin levels may reflect or mediate the wasting and malnutrition state that is common in this disease.

It is important to highlight that in CKD, additional to high adiponectin, low BMI has also been found to be related to increased all-cause and CV mortality [27]. Therefore, it has been hypothesized that the association of high adiponectin/low BMI could be the reflection of a process of protein–energy wasting (PEW). Among 1303 predialysis CKD patients, high adiponectin was found to associate independently with PEW [28]. Markaki et al. reported that in dialysis patients, adiponectin was also independently associated with PEW, and consequently with poor prognosis [29]. Moreover, Kaynar et al. reported that it is possible that adiponectin, as well as resistin, has a role in the development of PEW among dialysis patients [30].

Although enhanced adiponectin was associated with higher risk of death, it was not related to allograft failure in prevalent kidney transplant recipients [31]. Interestingly, a non-linear relationship between adiponectin and CVD was also reported, given that all-cause mortality and CVD occurrence risk was found to be higher in the lower and upper ranges of adiponectin value quadrants [32]. Controversially, in dialysis patients, an inverse association was found between adiponectin values and CV events and mortality; according to the authors, the inverse relationships of adiponectin with several metabolic risk factors (BMI, insulin levels, homeostatic model assessment index values, lipids, C-reactive protein (CRP) and left ventricular mass index (LVMI)) suggests a protective role of adiponectin in the prevention of CVD in patients under dialysis [33].

#### 2.2.2. Vascular Calcification

Vascular calcification is a common problem in ESRD. Studies in nephrectomized rats showed that globular adiponectin, the biologically active form of adiponectin with high affinity for AdipoR1 in the vascular smooth muscle cells (VSMC), protects these cells from calcification by inhibiting VSMC osteoblastic differentiation, probably by blocking the activation of the phosphatidylinositol 3-kinase (PI3K)/protein kinase B (AKT) and Wnt/β-catenin pathways, and the nuclear transcription of runt-related transcription factor 2 [34]. Liu et al. suggested that globular adiponectin suppresses vascular calcification through the inhibition of the mechanisms involved in the suppression of endoplasmic reticulum stress, which reduces cell apoptosis [35]. It was also suggested that this suppressive effect was induced by the Janus kinase 2 (JAK2)/signal transducer and activator of transcription 3 (STAT3) pathway, and that adiponectin may have an important role in the treatment of vascular calcification [36]. CKD patients with visceral obesity had higher coronary artery calcification scores and lower adiponectin levels compared to patients without visceral obesity [37].

Increased CV morbidity and mortality in dialysis patients is associated with vascular calcification. Artery calcification induces cardiovascular stiffening, leading to systolic hypertension, LVH and reduced coronary perfusion. Although calcium, phosphate and alkaline phosphatase have important roles in vascular calcification, and its association with abnormal lipid metabolism is known, the underlying mechanism remains unclear in CKD patients [38,39]. Curiously, according to Markaki et al., the predictive value of adiponectin regarding all-cause mortality in ESRD patients was observed only in subjects with low magnesium and high calcium serum levels [40].

Adiponectin has been reported to be implicated in CKD-associated aortic stiffness [41]. Sakura et al. found a positive association of circulating adiponectin with abdominal aortic calcification in Japanese male hemodialysis patients [42]. However, it was also reported that in patients with predialysis CKD, the prevalence of carotid arteriosclerosis was significantly higher in the group with low adiponectin than in the group with high adiponectin values [43]. Hypoadiponectinemia was also associated with aortic stiffness in non-dialysis patients at stages 3–5 CKD [44].

#### 2.2.3. Dyslipidemia

Patients with CKD frequently present dyslipidemia. In ESRD patients, increased triglyceride levels, normal or decreased values of total cholesterol and LDLc and decreased HDLc values are observed [9,45]. Our team reported that high adiponectin levels are important determinants of HDLc and triglyceride concentrations in CKD patients [46]. Additional to HDLc, adiponectin was also found to correlate positively with total cholesterol and LDLc [47]. Nonetheless, data regarding the relationships between adiponectin and the lipid profile are not consistent. For instance, regarding triglyceride levels, inverse correlations with adiponectin [47] and with the LMW isoform [48] were reported. A negative association of adiponectin with LDLc values was also observed [33].

**Figure 2:**
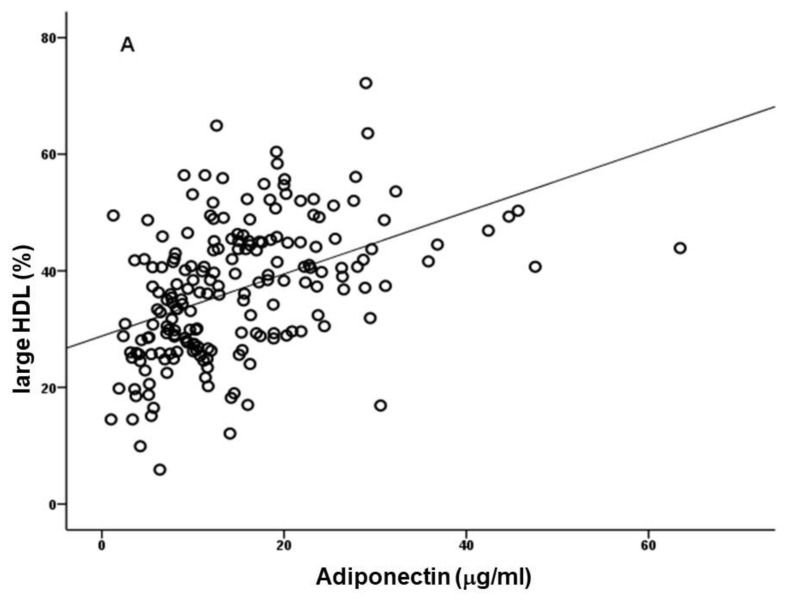
Association of circulating levels of adiponectin with large high-density lipoprotein (HDL), in end-stage renal disease patients under chronic hemodialysis (*r* = 0.509, *p* < 0.001). Adapted from Coimbra et al. 2019 [9].

A study on the lipoprotein subfractions in ESRD patients on dialysis suggested that adiponectin is positively correlated with large HDL (Figure 2) and inversely correlated with medium and small HDL; it appears that increased adiponectin leads to a more protective HDL profile [9]. Given the low levels of HDLc, we must not exclude the hypothesis that these differences in HDL profile (size and composition) may not have a relevant impact on CVD risk. The possibility that the alterations in size and composition are not accompanied by improvement in HDL functionality cannot be ruled out. Oxidized LDL (oxLDL) and the oxLDL/LDLc ratio showed a negative correlation with adiponectin; by performing a multiple linear regression analysis, we identified large HDL, oxLDL/LDLc ratio and BMI as the best predictors of adiponectin levels [9].

A study on the lipoprotein subfractions in ESRD patients on dialysis suggested that adiponectin is positively correlated with large HDL (Figure 2) and inversely correlated with medium and small HDL; it appears that increased adiponectin leads to a more protective HDL profile [9]. Given the low levels of HDLc, we must not exclude the hypothesis that these differences in HDL profile (size and composition) may not have a relevant impact on CVD risk. The possibility that the alterations in size and composition are not accompanied by improvement in HDL functionality cannot be ruled out. Oxidized LDL (oxLDL) and the oxLDL/LDLc ratio showed a negative correlation with adiponectin; by performing a multiple linear regression analysis, we identified large HDL, oxLDL/LDLc ratio and BMI as the best predictors of adiponectin levels [9].

#### 2.2.4. LVH

LVH is a common cardiovascular complication in CKD, especially in patients at stage 5, that has been positively associated with adiponectin levels [49] and with a negative prognostic value, contributing to diastolic dysfunction, congestive heart failure, arrhythmia and sudden death [50].

In ESRD patients on dialysis with type 2 diabetes mellitus, high adiponectin was also associated with LVH, and it was suggested that adiponectin levels could be modulated by a chronic hypervolemic state in these patients [51].

A positive association of adiponectin with IL-6, TNF-α and LVMI was also observed in dialysis patients, suggesting that the relationship between proinflammatory cytokines and adiponectin may have a role in the development of LVH in these patients [52].

It has also been proposed that the relationship between adiponectin and LVMI varies with the risk of LVH, since subjects at low risk of LVH showed an inverse association of adiponectin with LVMI, while a positive association was observed in subjects at high risk of LVH [53].

In children with CKD stage 2–4, higher adiponectin was associated with higher HMW and lower LMW isoforms, while decreased LMW adiponectin was found to be independently associated with higher LVMI [54]. Curiously, Abdallah et al. reported an inverse correlation between adiponectin and LVMI [33].

### 2.3. Mineral and Bone Disorder

Adiponectin is also an intervenient in mineral and bone disorders of patients with ESRD. High adiponectin levels have been associated with decreased bone mineral density in dialysis patients [55]. Moreover, adiponectin correlates positively with markers of bone disorders, such as bone alkaline phosphatase, tartrate-resistant acid phosphatase and undercarboxylated, carboxylated and intact osteocalcin; the last two markers are proven to be predictors of adiponectin concentrations [56].

Adiponectin is also correlated with parathyroid hormone (PTH) levels in various stages of CKD [41]. Considering that in adiponectin knockout mice, a decrease in PTH secretion was found after phosphate loading [57], and that phosphate retention is enhanced in CKD, this is an expected correlation.

Fibroblast growth factor (FGF)23, which is high in CKD, leads to an increase in phosphate excretion through the kidney proximal tubules, contributing to maintain the balance of phosphate metabolism. In renal patients with high adiponectin (but not low) levels, the high FGF23 correlates with vascular calcification, a common feature of CKD–mineral and bone disorder [58]. Furthermore, adiponectin was reported to be a strong modifier of the FGF23 response to vitamin D receptor activation in CKD patients [59].

### 2.4. Anemia

Anemia is a common complication in CKD, particularly in the latter stages, and the main cause is an inadequate production of erythropoietin by the failing kidneys. Several other factors may, however, contribute to the development/worsening of anemia, namely inflammation and the uremic milieu.

A prospective observational study, involving 1029 subjects of more than 40 years of age, evaluated the relationship between anemia and adiponectin; the study showed that high adiponectin levels were correlated with decreased erythroid-related variables, and thus proposed adiponectin as a risk factor for anemia [60]. Other studies, of elderly CKD patients, found that hemoglobin and estimated GFR were independently associated (inversely) with serum adiponectin [61,62]. In diabetic CKD patients, anemia was associated positively with high HMW adiponectin levels, independently of renal dysfunction [63].

The association of anemia with increasing levels of adiponectin is still not well understood. It has been proposed that the higher number of bone marrow adipocytes in CKD patients, as compared to healthy individuals, may contribute to higher circulating levels of adiponectin [64]; moreover, they were negatively correlated with hematopoiesis [65], suggesting a close relationship between adiponectin and the development of anemia in CKD patients. It is also known that chronic inflammation, dietary restriction, metabolic acidosis and several comorbid conditions are common in CKD patients, and contribute to the high prevalence of PEW in these patients; moreover, adiponectin is negatively correlated with BMI in these patients. Considering that protein malnutrition leads to bone marrow changes, compromising hematopoiesis, PEW might be another possible linkage between adiponectin and anemia in CKD patients.

It appears that the contribution of adiponectin to anemia in CKD patients might have different underlying factors beyond the traditional factors related to the disease, namely, aging, bone marrow changes, malnutrition, altered hematopoiesis and the profiles of adiponectin isoforms. Further studies are still need for a better understanding of this linkage.

### 2.5. Adiponectin Isoforms

The inconsistencies in data describing the role of adiponectin in different conditions may occur due to its complex and diversified actions. Moreover, the different physiological effects shown by the adiponectin isoforms may contribute to the inconsistent data on adiponectin.

Studies conducted with monocytic cells, which are common in adipose tissue, showed that LMW adiponectin induced anti-inflammatory effects, while the effects of HMW isoforms were proinflammatory [66,67]. The type of inflammatory response seems to be dependent on the predominant adiponectin isoform, and on the type of cell on which they act [68]. The activation of different signaling pathways may be another explanation for the different effects of the adiponectin isoforms. In innate immune cells, the pro- and anti-inflammatory responses to globular adiponectin are mediated by the activation of the nuclear factor- κB (NF-κB) pathway and IL-receptor-associated kinase-1 (IRAK-1) downregulation, respectively [69]. In vascular cells, HMW adiponectin activated AMPK and suppressed cytokine-induced NF-κB activation, indicating that HMW isoforms may have both anti- and proinflammatory actions [70]. Differences in tissue expression of adiponectin receptors may be another hypothesis to consider.

## 3. Leptin

Leptin is a 16 kDa globular protein that comprises 167 amino acids and presents a tertiary structure. It is mainly produced by adipocytes, and its levels are proportional to fat mass [71]. In blood, leptin circulates in free and protein-bound forms; biologically active leptin exists in the free form.

Its functions are mediated through leptin receptors (LepR) that exhibit structural similarity to the class I family of cytokine receptors. Six LepR isoforms, LepRa–f, have been identified, showing a common leptin binding domain and different intracellular domains. LepRa, b, c, d and f are transmembrane receptors that bind to leptin and activate JAK2; LepRe lacks a transmembrane domain and is a soluble isoform. Circulating leptin binds to soluble LepRe, resulting in the inhibition of central leptin transport. LepRb, a long isoform, is the most important receptor for the physiological effects of leptin, for energy homeostasis and for several other neuroendocrine functions [72]. Leptin activates phosphoinositol-3 kinase (PI3K) and mitogen-activated protein kinase (MAPK)/extracellular signal-regulated kinase (ERK) signaling pathways, which seems to contribute towards suppressing appetite, inducing weight loss and increasing thermogenesis [73]. The JAK/STAT3, MAPK/ERK and PI3K pathways appear to cooperate in the regulation of energy balance [74].

Leptin acts as a major regulator of food intake and energy homeostasis, reaching the brain by a saturable transport mechanism, through the blood brain-barrier. It influences several endocrine functions, bone metabolism and the stress response [75]. Leptin stimulates inflammation, oxidative stress, thrombosis, arterial stiffness, angiogenesis and atherogenesis [76]. It has several proinflammatory properties, for instance, it activates innate immune cells and stimulates the secretion of several proinflammatory cytokines, such as IL-6 and TNF-α. Conversely, TNF-α and IL-1 upregulates leptin production [75]. It also affects lipid and carbohydrate metabolism, blood pressure and hematopoiesis. Therefore, an increase in its production is frequently associated with the development of CVD.

Leptin also performs modulatory activities in innate and adaptive immune responses; when reduced, it is associated with higher risk of infection and diminished cell-mediated immunity.

Leptin is cleared from circulation by the kidneys through glomerular filtration and metabolic degradation in renal tubules. Hyperleptinemia has direct and indirect negative effects on renal function [77]. Development of CVD, thickening of the basement membrane of the proximal tubular cells (which increase protein leakage into filtrate) and the activation of protein synthesis (which favors fibrosis and glomerulosclerosis), of NADPH oxidase (which increases reactive oxygen species production and, consequently, inflammation), of the sympathetic nervous system in the kidneys and of the renin–angiotensin–aldosterone system (associated with sodium retention, predisposing it to high blood pressure) are some potential mechanisms proposed to explain the damaging effect of hyperleptinemia on renal function [78].

Circulating leptin concentrations are enhanced in patients with CKD, and are associated with the progression of renal deterioration, which could be, in part, assigned to the compromise of renal clearance [79]. Leptin is not removed by dialysis procedures using low-flux membranes; however, hemodialysis and hemodiafiltration using high-flux membranes are able to reduce circulating leptin [80,81].

Leptin, and the leptin to adiponectin ratio, has been associated inversely with peritoneal creatinine clearance in patients that had recently started peritoneal dialysis [82].

### 3.1. Inflammation and Leptin

Inflammation, a hallmark of CKD, is frequently associated with hyperleptinemia once, as already referred to, proinflammatory cytokines stimulate leptin production.

Visceral adipocytes exposed to the uremic plasma of ESRD patients presented an enhanced release of leptin that was linked to an avid uptake of TNF-α by the adipocytes, emphasizing the role of TNF-α in the production and release of leptin in ESRD [83]. Leptin, and the leptin/adiponectin ratio, correlated positively with TNF-related apoptosis-inducing ligands, known to induce apoptosis, although it can also activate antiapoptotic signals [84].

In CKD patients, leptin, IL-6, TNF-α and adiponectin are significantly increased, and increase with CKD severity [84]. Leptin has also been positively correlated with CRP in CKD, increasing both with progression of the disease [85].

By inducing the production of proinflammatory mediators and reactive oxygen species [86,87,88], leptin may also contribute to the inflammatory process found in renal disease and, thereby, to CVD complications.

### 3.2. CVD Risk and Leptin

#### 3.2.1. BMI

A high BMI is one of the strongest risk factors for new-onset CKD [89]. Obesity seems to affect the kidneys via the endocrine activity of the adipose tissue, with increased production of adipokines, such as leptin.

Metabolic syndrome refers to the coexistence of obesity, insulin resistance, atherogenic dyslipidemia and hypertension. In children at stages 2–4 of CKD, leptin was found to correlate positively with BMI and triglyceride levels, and negatively with HDLc and insulin resistance [90]. It has been suggested that in ESRD patients, high leptin concentrations might be a consequence of metabolic syndrome, which is highly prevalent in ESRD [91]. A study by Tsai et al. of hemodialysis patients showed that fasting leptin correlated positively with metabolic syndrome, and that pre-hemodialysis body weight was a possible influencer of leptin levels in these patients [92]. It was also reported that the predictive value of leptin for all-cause and CV death seems to be dependent on waist circumference [93], one of the criteria used to define metabolic syndrome. According to our data, patients on dialysis with diabetes mellitus and patients with both diabetes mellitus and hypertension had higher leptin levels than those without diabetes mellitus or hypertension, or only with hypertension [9]. Controversially, others have reported no significant associations between leptin or the leptin/BMI ratio and all-cause and CVD-related mortality in patients on hemodialysis (HD) [94].

On the other hand, dialysis patients with a higher BMI showed better nutritional status compared to normal or overweight subjects [95], suggesting a role for fat in nutritional status. It was reported that, in dialysis patients, subcutaneous fat may be an indicator of nutritional status, while visceral fat is probably an indicator of inflammation [96]. Non-obese patients under hemodialysis with elevated leptin concentrations also presented a good nutritional status [97], suggesting that high BMI may not influence nutritional status.

ESRD is frequently associated with anorexia, malnutrition and hypervolemia, a setting that seems to correlate with leptin levels. In dialysis patients, leptin was independently associated with PEW and, consequently, with poor prognosis [29]. In a study conducted on hemodialysis subjects, the deceased patients presented lower leptin values, which were associated with hypervolemia and malnutrition [98]. In patients under dialysis, hypervolemic subjects presented lower leptin values and poorer nutritional status than normovolemic patients [99]; overhydrated dialysis patients also presented lower leptin than normohydrated patients [100]. Considering the possible influence of dialysis in the relationship between leptin–malnutrition–hypervolemia, patients with stage 5 CKD who were not undergoing dialysis were studied, and it was found that those with poor nutritional status also suffered from excessive body fluid and presented lower leptin levels [101].

However, dialysis patients with diabetes mellitus and malnutrition–inflammation–atherosclerosis syndrome presented higher leptin levels, and lower HMW adiponectin, than non-diabetic patients with malnutrition–inflammation–atherosclerosis syndrome [102], indicating a relationship between the existence of diabetes and enhanced leptin levels.

#### 3.2.2. Vascular Complications

CKD patients diagnosed with visceral obesity had higher scores for coronary artery calcification and leptin levels, as compared to patients without visceral obesity [37]. Vascular calcification is a regulated and complex process involving abnormal cell transitions and osteogenic differentiation, readapting of signaling pathways to those occurring in bones, and, eventually, with the formation of osteoclast-like cells; endothelial cells have been shown to contribute to vascular calcification [103]. Studies of CKD patients and in vitro data on human umbilical vein endothelial cells indicate that the higher leptin concentrations promote endothelial dysfunction in CKD [104,105]. In vitro studies showed that leptin activates the AKT/GSK3β/β-catenin pathway, increasing the levels of ICAM (intercellular adhesion molecule)-1 and VCAM (vascular cell adhesion molecule)-1 and the rearrangement of the cytoskeleton, which results in increased endothelial cell migration and enhanced monolayer permeability, thus, favoring endothelial dysfunction [104]. In CKD patients, leptin levels were found to correlate positively with the circulating soluble forms of ICAM-1 and VCAM-1 [104]. In patients with stage 3–5 CKD, leptin levels also correlated positively with aortic stiffness [106], known to be connected to arterial media calcification [107].

#### 3.2.3. Dyslipidemia

In CKD, leptin and leptin/BMI were found to be independent predictors of total cholesterol and triglyceride values, being associated with a more atherogenic lipid profile [108]. In children with CKD stages 2 to 4, leptin levels associated positively with triglyceride values and negatively with HDLc concentrations [90]. The HDL/LDL ratio was lower in CKD patients as compared to the control group, and correlated with leptin, which suggested that hyperleptinemia observed in CKD contributes to pathogenesis of CVD by decreasing HDL/LDL ratio [85]. Furthermore, and as already mentioned, atherogenic dyslipidemia is a component of metabolic syndrome, the occurrence of which is associated with high leptin levels [91]. It was also reported that CKD-related alterations of the fatty acid profile may contribute to elevated serum leptin concentrations in patients with CKD by increasing its gene expression in subcutaneous adipose tissue [109].

#### 3.2.4. LVH

Surprisingly, in ESRD, lower leptin levels have been associated with a poorer prognosis regarding CV events and mortality. Scholze et al. reported that low leptin levels were an independent predictor of mortality in ESRD patients under hemodialysis [110]. Even in prevalent kidney transplant recipients, lower leptin was found to be an independent predictor of death [111]. In maintenance hemodialysis patients, decreased circulating levels of leptin were associated with a higher risk of CV events and death, probably contributing to LVH and peripheral vascular disease development [112]. Qin et al. described that decreased leptin levels were an independent risk factor for LVH development in patients on hemodialysis [112]. Curiously, in ESRD, patients with a history of stroke presented higher leptin levels than those without stroke history, while patients with congestive heart failure showed lower leptin values that those without history of congestive heart failure [113].

### 3.3. Mineral and Bone Disorder

Leptin appears to stimulate osteoblastic proliferation and differentiation and to inhibit adipogenic differentiation from marrow stromal cells; however, as well as positive, also both negative and no effects of leptin in bone mass have been described [114]. In ESRD patients, bone mineral density is associated with body composition, in particular total fat mass, nutritional status and mortality risk [115]. Leptin levels were found to correlate inversely with markers of bone turnover and PTH, which led to hypothesize that leptin lowers bone turnover in ESRD [116]. Wang et al. found that increased leptin, body weight and serum albumin were positively related to bone mineral density in hemodialysis patients [117]. Leptin was reported to have a bone-sparing effect in hemodialysis patients, but only when its serum levels were higher than the presumed threshold of blood–brain transport saturation [118]. However, PTH, in addition to BMI, insulin and metabolic syndrome score, was found to be an independent predictor of leptin values with both, PTH and leptin, correlating positively in patients at different CKD stages [119]. Additionally, it was found that in 29 female hemodialysis patients, leptin levels were lower in those with PTH > 300 pg/mL, bone alkaline phosphatase between 300–600 IU/L and calcium < 8.5 mg/dL, as compared to female with PTH between 100–300 pg/mL, bone alkaline phosphatase < 300 IU/L and calcium between 8.5–10.5 mg/dL, respectively; these results were not found for male patients or when considering all the 73 patients studied, and the values of these biomarkers did not differ significantly between the two genders [120].

### 3.4. Anemia

An independent association between lower leptin levels and anemia was found, and in stage 5 CKD patients that were submitted to parathyroidectomy, increased leptin was associated with ameliorated anemia and malnutrition [121].

In CKD, leptin levels were identified as possible predictors of epoetin sensitivity; in the presence of high leptin levels, proinflammatory cytokines appear not to have inhibitory effects on epoetin sensitivity [122]. Hyperleptinemia was reported to better reflect recombinant human erythropoietin response and nutritional status in long-term dialysis patients [123]. However, it was reported that dialysis patients with diabetes and malnutrition–inflammation–atherosclerosis syndrome had significantly higher leptin levels and required higher erythropoietin dose; indeed, erythropoietin dosage was associated significantly with levels of leptin and biomarkers of inflammation [102].

In ob/ob mice, circulating hepcidin concentrations were found to be significantly lower as compared to control group; after leptin administration, hepcidin levels and liver expression of Hamp mRNA increased [124]. Leptin seems to favor the enhancement of hepcidin, the major regulator of iron metabolism, since it inhibits iron intestinal absorption and reduces iron mobilization from macrophages of the reticuloendothelial system.

As for adiponectin, data concerning the impact of leptin levels on CKD comorbidities are not always consistent. Conflicting results may be related to renal replacement therapy used, for instance, high-flux hemodialysis and hemodiafiltration were shown to considerably reduce leptin levels [80,81]. In CKD, the coexistence of complications, such as diabetes mellitus, obesity, metabolic syndrome, and hypervolemia, may also contribute to the contradictory data.

## 4. Concluding Remarks

Paradoxically, the levels of both adiponectin and leptin, known, respectively, for their anti- and proinflammatory effects, and for their negative and positive correlation with BMI, are elevated in CKD, and have been associated with progression of renal dysfunction.

**Figure 3:**
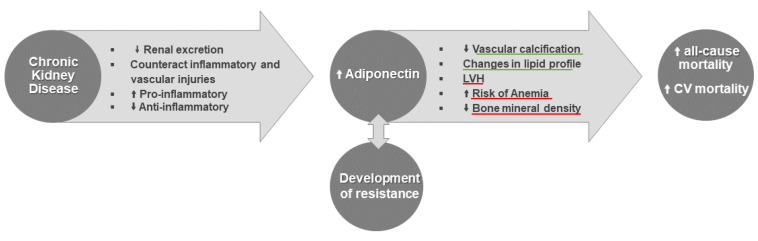
Schematic view of the hypotheses raised to explain the high adiponectin concentrations found in chronic kidney disease (CKD) and of the most likely effects, negative (underlined in red) and positive (underlined in green), of hyperadiponectinemia that may contribute to the high all-cause and cardiovascular (CV) mortality rates found in CKD patients. LVH, left ventricular hypertrophy. ↑ indicates increase, ↓ indicates decrease.

According to the data collected to date, in CKD, the increase in adiponectin associates with decreased bone mineral density, development of anemia and LVH; however, beneficial associations, namely, in vascular calcification and lipid profile, have been also reported (Figure 3). It should be highlighted that data concerning adiponectin in CKD is not always consistent, probably due to its complex and diversified actions. In addition, adiponectin isoforms and their different roles, the activation of different signaling pathways and the different tissue expression of its receptors, may also contribute to that controversial data.

**Figure 4:**
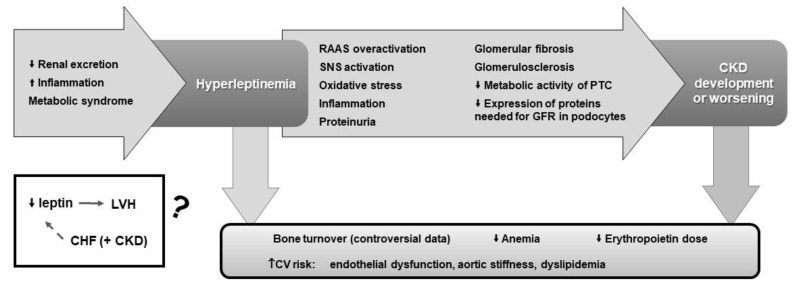
Schematic view of the causes that favor the increase in leptin levels in chronic kidney disease (CKD), of the negative actions of hyperleptinemia in renal function, that favors CKD development or worsening, and of its relations with common CKD comorbidities. An appointment to the surprising relation of low leptin levels and the occurrence of left ventricular hypertrophy (LVH), which may be related to the coexistence of congestive heart failure (CHF) in CKD patients, is also presented. CV, cardiovascular; GFR, glomerular filtration rate; PTC, proximal convoluted tubule cells; RAAS, renin-angiotensin-aldosterone system; SNS, sympathetic nervous system. ↑ indicates increase, ↓ indicates decrease.

In CKD, hyperleptinemia has been associated with endothelial dysfunction, aortic stiffness and dyslipidemia, all known as risk factors for CVD (Figure 4). Its increase has also been related to a decrease in the risk of developing anemia but, eventually, with a higher erythropoietin dose requirement in case of anemia. Surprisingly, in ESRD, lower leptin levels were found to be an independent risk factor for LVH, which may be related to the coexistence of congestive heart failure. It is important to recall that in ESRD patients, leptin levels are dependent on the volemia state and the type of renal replacement used.

Controversial changes in the levels of these adipokines in CKD patients, as reported in the literature, may also reflect the coexistence of several comorbidities, such as diabetes, hypertension, metabolic syndrome and malnutrition, among others.

Metabolic syndrome, associated with insulin resistance, weight gain, hypertension, dyslipidemia and inflammation, is a common CKD comorbidity. Leptin and adiponectin have opposite impacts on insulin resistance and inflammation. With increasing of weight, adipose tissue becomes dysfunctional, resulting in hyperleptinemia and, eventually, in resistance to leptin and its effects. In type 2 diabetes, insulin resistance was found to correlate with leptin levels [125,126]; it is possible that the hyperleptinemia found in these subjects reflects leptin resistance. It was proposed that hyperinsulinemia favors obesity, contributing to a higher increase in leptin [126,127,128]. Moreover, hyperleptinemia, due to weight gain, appears to deregulate the control of blood pressure, favoring hypertension [126,127,128]. Leptin stimulates the production of proinflammatory cytokines and, consequently, of acute-phase reactants; thus, hyperleptinemia potentiates inflammation. On the other hand, adiponectin levels decrease with increasing BMI, being lower in overweight and obese subjects. Hypoadiponectinemia favor insulin resistance and type 2 diabetes [129], and predisposes to hypertension [130].

The occurrence of malnutrition–inflammation–atherosclerosis syndrome is common in ESRD. In CKD patients on dialysis treatment, leptin correlated inversely with malnutrition–inflammation score, while adiponectin presented a weak positive correlation, suggesting an association between the increase in adiponectin with a worse nutritional status [131]. In patients with malnutrition–inflammation–atherosclerosis syndrome and type 2 diabetes, compared to patients with this syndrome but without diabetes, higher levels of leptin (and IL-6 and hs-CRP) and lower HMW adiponectin values were observed [102]. The levels of these adipokines are influenced when different comorbidities exist.

It seems that adiponectin and leptin contribute to a higher risk of CVD events and mortality in CKD patients, but further studies are warranted to fully clarify their roles, especially when different comorbidities exist.

## Figures and Tables

**Table 1:** Adiponectin and leptin properties.

	Adiponectin	Leptin
Main site of synthesis	Adipocyte	Adipocyte
Molecular weight	30 kDa	16 kDa
Receptors	AdipoR1, AdipoR2 and T-cadherin	LepR (six isoforms: a–f)
Biological actions related to CVD	Anti-inflammatory Antidyslipidemic Antidiabetic Changes in arterial stiffness	Regulation of food intake and insulin sensitivity Proinflammatory Changes in arterial stiffness
Circulating levels in ESRD	↑↑	↑

AdipoR, adiponectin receptor; CVD, cardiovascular disease; ESRD, end-stage renal disease; LepR, leptin receptor. ↑ and ↑↑ indicate high and very high circulating adiponectin/leptin in ESRD respectively.

## Data Availability

Data sharing is not applicable to this article as no new data were created or analyzed in this study.
